# Adequacy of control of cardiovascular risk factors in ambulatory patients with type 2 diabetes attending diabetes out-patients clinic at a county hospital, Kenya

**DOI:** 10.1186/s12902-017-0223-1

**Published:** 2017-12-01

**Authors:** Mercy W. Kimando, Frederick C. F. Otieno, Elijah N. Ogola, Kenn Mutai

**Affiliations:** 1 Department of Clinical Medicine and Therapeutics, College of Health Sciences, University of Nairobi, Nairobi, Kenya; 2 Kenyatta National Hospital, Nairobi, Kenya

## Abstract

**Background:**

Type 2 diabetes is associated with substantial cardiovascular morbidity and mortality arising from the high prevalence of cardiovascular risk factors such as hypertension, dyslipidaemia, obesity, poor glycaemic control and albuminuria. Adequacy of control of these risk factors determines the frequency and outcome of cardiovascular events in the patients. Current clinical practice guidelines emphasize primary prevention of cardiovascular disease in type 2 diabetes. There is scarce data from the developing countries, Kenya included, on clinical care of patients with type 2 diabetes in the regions that are far away from tertiary health facilities. So we determined the adequacy of control of the modifiable risk factors: glycaemic control, hypertension, dyslipidemia, obesity and albuminuria in the study patients from rural and peri-urban dwelling.

**Methods:**

This was a cross-sectional study on 385 randomly selected ambulatory patients with type 2 diabetes without overt complications. They were on follow up for at least 6 months at the Out-patient diabetes clinic of Nyeri County Hospital, a public health facility located in the central region of Kenya.

**Results:**

Females were 65.5%. The study subjects had a mean duration of diabetes of 9.4 years, IQR of 3.0–14 years. Their mean age was 63.3 years, IQR of 56-71 years.

Only 20.3% of our subjects had simultaneous optimal control of the three (3) main cardiovascular risk factors of hypertension, high LDL-C and hyperglycaemia at the time of the study. The prevalence of cardiovascular risk factors were as follows: HbA1c above 7% was 60.5% (95% CI, 55.6–65.5), hypertension, 49.6% of whom 76.6% (95% CI, 72.5–80.8) were poorly controlled. High LDL-Cholesterol above 2.0 mmol/L was found in 77.1% (95% CI 73.0–81.3) and Albuminuria occurred in 32.7% (95% CI 27.8–37.4). The prevalence of the other habits with cardiovascular disease risk were: excess alcohol intake at 26.5% (95% CI 27.8–37.4) and cigarette-smoking at 23.6%.

A modest 23.4% of the treated patients with hypertension attained target blood pressure of <140/90 mmHg. Out of a paltry 12.5% of the statin-treated patients and others not actively treated, only 22.9% had LDL-Cholesterol of target <2.0 mmol/L.

There were no obvious socio-demographic and clinical determinants of poor glycaemic control. However, old age above 50 yrs., longer duration with diabetes above 5 yrs. and advanced stages of CKD were significantly associated with hypertension. Female gender and age, statin non-use and socio-economic factor of employment were the significant determinants of high levels of serum LDL-cholesterol.

**Conclusion:**

The majority of the study patients attending this government-funded health facility had high prevalence of cardiovascular risk factors that were inadequately controlled. Therefore patients with type 2 diabetes should be risk-stratified by their age, duration of diabetes and cardiovascular risk factor loading. Consequently, composite risk factor reduction strategies are needed in management of these patients to achieve the desired targets safely. This would be achieved through innovative care systems and modes of delivery which would translate into maximum benefit of primary cardiovascular disease prevention in those at high risk. It is a desirable quality objective to have a higher proportion of the patients who access care benefiting maximally more than the numbers we are achieving now.

## Background

The prevalence of cardiovascular disease is strikingly increased in persons with diabetes more than those without diabetes [1]. Cardiovascular events make about eighty (80%) percent of the morbidity and mortality in the patients with type 2 diabetes [2].

Type 2 diabetes mellitus is often co-morbid with the cardiovascular risk conditions that include: modifiable ones, being hypertension, dyslipidemia, obesity, smoking and poor glycemic control, and the non-modifiable ones of aging and genes that have been associated with enhanced cardiovascular morbidity. The developed world has experienced improved care and outcomes in patients with type 2 diabetes but quite a high proportion of treated patients have not achieved desired targets in glucose, blood pressure and cholesterol control [3].

Sub-Saharan Africa, like the rest of the world, is experiencing an increasing prevalence of diabetes alongside other non-communicable diseases. The prevalence of diabetes ranges from.

4.3% in sub-Saharan Africa, 6.7% in Europe, 10.5% in North America and the Caribbean to 10.9% in the Middle East and North Africa [4]. These numbers project the care demands of people with type 2 diabetes now and in the future that sub-Saharan Africa may not be adequately prepared for.

The INTERHEART study, which included participants from sub-Saharan Africa, identified nine cardiovascular risk factors, (diabetes, hypertension, dyslipidaemia, smoking, obesity, unhealthy diet, physical inactivity, alcohol consumption, psychosocial stresses), that explained more than 90% of the coronary events in the study [5]. That the prevalence of cardiovascular risk factors in type 2 diabetes is high, and the consequences of clinical events is burdensome to patients, their families, and society cannot be overemphasized.

Factors that affect optimal control of these risk factors include access to care, cost of medication and care, socio- economic factors at national and individual levels and psychosocial support system [6]. Sustained adequate cardiovascular risk factor control in a high proportion of patients with type 2 diabetes remains elusive. Therefore more studies are needed, especially in resource-constrained settings, to evaluate the care provision for Quality Improvement, to determine the proportions of patients not attaining targets and underlying reasons to intervene on. This study was conducted to audit the care provided to patients with type 2 diabetes in the public health facility.

## Methods

### Study design and population

This was a cross-sectional study conducted over 4-month period between December 2014 and March 2015 at the diabetes out-patient clinic in Nyeri level 5 hospital, a public health facility. About 7000 patients with diabetes (both types 1 and 2, old and new) were seen in the previous calender year. This was an audit of the clinical care delivered to the patients with type 2 diabetes at this health facility.

This clinic is held once-weekly on Fridays except on public holidays. It has dedicated staff of a Medical Officer, Nurses and an Educator who is a dietitian, but not specifically trained in diabetes care. Their activities at the clinic include weight and height measurements, Blood Pressure determination, and random blood glucose (after the patient has paid for this test). There are only a very small number of patients who perform self-monitoring of blood glucose. Diabetes Education for Self-Management is offered to the clinic attendees as a group, individual approach is only an occasional encounter.

**Fig. 1:**
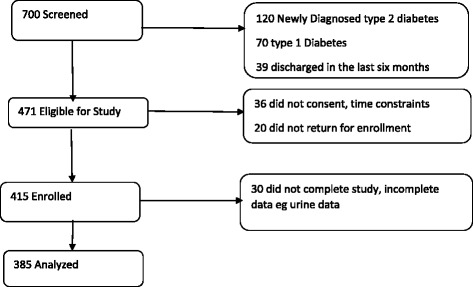
A flow chart of subject recruitment, enrolment into and activities in the study

The participants targeted were patients with file diagnosis of type 2 diabetes, previously diagnosed the standard way by the primary physician. The patients were randomly selected from amongst the clinic attendees of the day. Those included in the study were aged 30 years and above, on follow-up for at least 6 months, on either oral anti-diabetic medication alone or in combination with insulin or diet-only. The flow chart, Fig. 1 **,** below depicts the enrolment process.

### Ethics, consent and approval

**Fig. 2:**
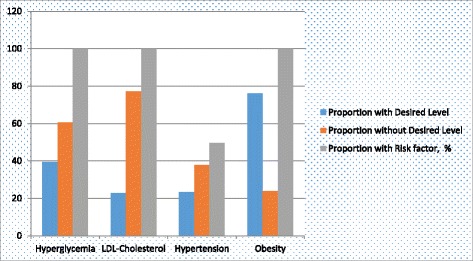
Bar Chart depicting selected Cardiovascular Risk Factors in control and the number at risk amongst the study subjects

The study was approved by the Depart of Clinical Medicine and Therapeutics and the Ethics Review Committees of UoN/KNH and of the Nyeri County hospital. Full explanation was given to the eligible patient and informed written consent was obtained from each subject before enrolment (Fig. 2).

### Study assessments and clinical methods

A complete history was taken from each study participant for socio-demographic information, relevant clinical information on the diabetes mellitus including its treatment and any hypertension or cardiovascular disease. Social habits of alcohol intake and cigarette smoking were documented. The age was verified with patient’s national identity card. The marital status was recorded as reported by the study patient. The last prescription and/or the file notes of the last review was used to corroborate the treatment information. Full clinical examination was performed.

Blood pressure was measured by the doctor after the study participant had rested for about 10-min from the time of arrival. While seated with the arm in a comfortable position at the level of the heart, systolic blood pressure was taken at the 1st Korotkoff sound and the diastolic blood pressure taken at the disappearance of Korotkoff sound on a manual mercury sphygmomanometer, both values were measured to the nearest mmHg. The presence of hypertension was taken at BP ≥140/90 mmHg, as classified per JNC 8 [7]. Waist and Hip circumferences were measured in centimeters on the transverse plane at the level of the narrowest part of torso between the lowest rib and the top of pelvis as seen on the anterior view at the end of normal expiration for waist circumference. This was at the level of greater trochanter on the transverse plane for hip circumference. Waist hip ratio (WHR) was calculated as a ratio of Waist Circumference to Hip Circumference [8] and classified as per the NCEP/ATP III guidelines respectively [9]. Height was measured on the patient standing without shoes, the back against the wall on tape, to the nearest ten centimeter. Weight was taken on an electronic weighing machine to the nearest 0.5 kg. Body mass index (BMI) was calculated as weight in kilograms divided by height in meters squared and classified [10].

### Laboratory methods

The patients were advised to come to the diabetes clinic day, every Friday morning fasted. An 8 ml- venous blood sample was asceptically drawn from the cubital fossa. A six (6) ml-sample was collected in clot-activated vacutainers for serum lipid profile and the remaining 2 ml-sample into EDTA vacutainer for HbA1c determination. The samples were stored in cool boxes with dry ice carbon dioxide at 2 to 8 °C, then delivered to the laboratory of the University’s Department of Clinical medicine for assays later. HBA1c was processed by glycohemoglobin ion exchange resin method from *ERBA MANNHEIM Gmbh* at the laboratory. HbA1c > 7.0% was considered sub-optimal control.

Lipid profile was analyzed using *Human Gmbh* kit. Total cholesterol was measured using the CHOD-PAP method based on Trinders Methodology, a calorimetric, enzymatic test for cholesterol with lipid clearing factor. HDL cholesterol was measured using human cholesterol liqui-color Phosphatungstic Acid method, end-point kit. Triglycerides were measured using *GPO-PAPA METHOD*, a colorimetric, enzymatic method with glycerophosphate oxidase*.* LDL-cholesterol was computed from the formula: [LDL-chol] = [Total chol] - [HDL-chol] - ([TG]/2.2) where all concentrations are given in mmol/L. LDL-Cholesterol above 2.0 mmol/L was considered high.

#### Urinary albumin

Creatinine ratio was determined using the *CLINITEK Microalbuminuria reagent strips.* CLINITEK Microal-2 Strips provided albumin-to-creatinine ratio results in one minute once the strip was placed in an analyzer after being dipped in urine. Estimated glomerular filtration rate was calculated on Cockroft-Gault formula [11].

#### Data management and statistics

The data collected were entered into a spreadsheet and cleaned before analysis. Statistical analysis was done in SPSS version 21.0. Descriptive data such as socio-demographic (age, gender, marital status, level of education and employment status) and clinical characteristics (treatment information, Blood Pressure, categories of metabolic control) were summarized into percentages and means/medians. Prevalence of cardiovascular risk factors were presented as proportions of the total number of patients studied or with the risk factor. In addition, variables associated with the cardiovascular risk factors were analyzed. All the associations/comparisons were determined using Chi-square/Fisher’s exact test for categorical variables and Student t-test for comparison of means. Odds ratios at 95% confidence intervals (CIs) were calculated as estimates of relative risks of having poor control of cardiovascular risk factors among patients. Logistic regression model was used to determine independent predictors. Statistical significance was interpreted at a *p*-value of less than 0.05.

## Results

We enrolled 385 patients with type 2 diabetes into the study. The baseline characteristics are presented in the Tables 1, 2, 3 and 4. The key cardiovascular risk factors were: Hypertension, Obesity, high LDL-cholesterol, Hyperglycaemia and Albuminuria.

**Table 1:** Clinical and laboratory characteristics of the study subjects

Variable	Frequency (%)
BMI, mean (SD), kg/m^2^	26.7 (4.6)
Categories, n (%)	
Underweight (<18.5)	6 (1.6)
Normal (18.5–25)	139 (36.1)
Overweight (25–29.9)	154 (40.0)
Obese (≥30)	86 (22.3)
Blood Pressure (BP) mmHg	
Hypertensive, BP > 140/90 mmHg or on treatment	191 (49.6)
Normal BP	194 (50.4)
Hypertension treatment, Users,	
ACEi’s/ARBs	132 (69.4)
Calcium channel blocker(CCBs)	120 (63.0)
Diuretics(thiazides, Spironolactone, Furosemide)	90 (47.8)
Glucose-lowering treatment	
Diet-only	11 (2.9)
Oral Glucose-lowering Agents(OGLAs)-only	262 (68.1)
Insulin-only	66 (17.1)
Combined Oral Glucose-lowering Agents and Insulin	46 (12.0)
Glycemic control	
Mean HbA1c, %	8.3(3.0)
Poor (HbA1c > 7%)	233 (60.5)
Good (HbA1c ≤ 7%)	152 (39.5)
Total cholesterol, mean (SD), mmol/L	4.6 (1.2)
Categories, n (%)	
High ≥4.12	88 (22.9)
Normal, <4.12	297 (77.1)
HDL, mean (SD), mmol/L	1.3 (0.9)
Categories, n (%)	
Low ≤1.00	80 (20.8)
Normal >1.00	305 (79.2)
Triglycerides, mean (SD), mmol/L	1.7 (1.0)
Categories, n (%)	
High, >1.7	210 (54.5)
Normal ≤1.7	175 (45.5)
LDL, mean (SD), mmol/L	2.4 (0.9)
Categories, n (%)	
High, >2.0	297 (77.1)
Normal ≤2.0	88 (22.9)
Albuminuria	
Albuminuria	126 (32.7)
Normal	259 (67.3)

The socio-demographic characteristics of the 385 patients who were included in the study are shown in Table 2 below

**Table 2:** Socio-Demographic characteristics of the study patients

Variable	Overall *n* = 385(100%)	Female *n* = 252(65.5%)	Male *n* = 133(34.5%)
Mean age (SD) years	63.3 (12.1)	62.1 (12.0)	65.7 (11.8)
Marital status			
Married	258(67.7)	138 (54.8)	120 (45.2)
Separated	3(0.8)	2 (0.8)	1 (0.8)
Single, unmarried	22(5.7)	19 (7.5)	3 (2.3)
Widowed	102 (26.5)	93 (36.9)	9 (6.8)
Level of Formal Education			
None	51 (13.2)	48 (19.0)	3 (2.3)
Primary (1–7 years in school)	227 (59.0)	157 (62.3)	70 (52.6)
Secondary (8–12 years)	90 (23.4)	40 (15.9)	50 (37.6)
Tertiary (above 12 years)	17 (4.4)	7 (2.8)	10 (7.5)
Employment Status			
Employed	172(44.7)	123(48.8)	49(36.8)
Unemployed	213(55.3)	129(51.2)	84(63.2)

There was a female preponderance at 65.5% and more than 70% of them had either no formal education or a modest one of primary school level

**Table 3:** Prevalence of selected cardiovascular risk factors in the study subjects

Cardiovascular Risk Factors	Prevalence	95% CI
Poor Glycemic control, HbA1c ›7.0%	60.5%	55.6–65.5
Poorly controlled Hypertension, BP ≥ 140/90 mmHg	76.6%	72.5–80.8
LDL Cholesterol, >2.0 mmol/L	77.1%	73.0–81.3
Obesity, BMI ≥30 kg/m^2^	22.3%	18.4–26.5
Obesity, Waist Circumference	58.2%	53.5–62.9
>102 cm males		
> 88 cm females		
Obesity, Waist – Hip Ratio	92.7%	89.9–95.3
>0.9 males		
>0.8 females		
Albuminuria	32.7%	27.8–37.4
Cigarette-smoking, self-reported	23.6%.	19.9–28.5

The prevalence of uncontrolled hypertension, high LDL-Cholesterol and obesity (by WHR) was high, above 75% as shown in the table above

**Table 4:** Logistic regression of factors influencing the cardiovascular risk factor control in the study subjects

Variable	Glycemic control			Hypertension			LDL-Cholesterol			Smoking		
	Poor control	OR (95% CI)	*P* value	Hypertensive	OR (95% CI)	P value	High LDL	OR (95% CI)	P value	Smoker	OR (95% CI)	P value
Age												
> 50 years	198 (59.8)	0.8 (0.4–1.5)	0.486	165(49.8)	4.7 (2.6–8.5)	<0.001	251 (75.8)	0.5 (0.2–1.2)	0.129	84 (25.4)	2.0 (0.9–4.3)	0.091
< =50 years	35 (64.8)	1.0		26 (48.1)	1.0		46 (85.2)	1.0		8 (14.8)	1.0	
Gender												
Female	154 (61.1)	1.1 (0.7–1.6)	0.744	119 (47.2)	0.8 (0.5–1.2)	0.197	206 (81.7)	2.1 (1.3–3.4)	0.003	2 (0.8)	0.0 (0.00–0.02)	<0.001
Male	79 (59.4)	1.0		72 (54.1)	1.0		91 (68.4)	1.0		90 (67.7)	1.0	
Marital status												
Married	153 (59.3)	1.0		132 (51.2)	1.0		198 (76.7)	1.0		83 (32.2)	1.0	
Separated	2 (66.7)	1.4 (0.1–15.3)	0.797	2 (66.7)	1.9 (0.2–21.3)	0.599	2 (66.7)	0.6 (0.1–6.8)	0.685	1 (33.3)	1.1 (0.1–11.8)	0.966
Single, unmarried	16 (72.7)	1.8 (0.7–4.8)	0.222	9 (40.9)	0.7 (0.3–1.6)	0.359	19 (86.4)	1.9 (0.5–6.7)	0.307	2 (9.1)	0.2 (0.0–0.9)	0.039
Widowed	62 (60.8)	1.1 (0.7–1.7)	0.796	48 (47.1)	0.8 (0.5–1..3)	0.483	78 (76.5)	1.0 (0.6–1.7)	0.956	6 (5.9)	0.1 (0.1–0.3)	<0.001
Education												
None	31 (60.8)	1.0		27 (52.9)	1.0		41 (80.4)	1.0		3 (5.9)	1.0	
Primary	138 (60.8)	1.0 (0.5–1.9)	0.999	112 (49.3)	0.9 (0.5–1.6)	0.642	179 (78.9)	0.9 (0.4–1.9)	0.807	49 (21.6)	4.4 (1.3–14.7)	0.016
Secondary	54 (60.0)	1.0 (0.5–2.0)	0.927	45 (50.0)	0.9 (0.4–1.8)	0.737	69 (76.7)	0.8 (0.3–1.9)	0.608	35 (38.9)	10.2 (2.9–35.2)	<0.001
Tertiary	10 (58.8)	0.9 (0.3–2.8)	0.886	7 (41.2)	0.6 (0.2–1.9)	0.403	8 (47.1)	0.2 (0.1–0.7)	0.011	5 (29.4)	6.7 (1.4–31.9)	0.017
Employment status												
Unemployed	78 (62.4)	1.0		65 (52.0)	1.0		95 (76.0)	1.0		15 (12.0)	1.0	
Employed	14 (60.9)	0.9 (0.4–2.3)	0.889	8 (34.8)	0.5 (0.2–1.2)	0.134	17 (73.9)	0.9 (0.3–2.5)	0.830	4 (17.4)	1.5 (0.5–5.2)	0.480
Self-employed	88 (59.1)	0.9 (0.5–1.4)	0.573	69 (46.3)	0.8 (0.5–1.3)	0.348	124 (83.2)	1.6 (0.9–2.8)	0.139	27 (18.1)	1.6 (0.8–3.2)	0.164
Retired	53 (60.2)	0.9 (0.5–1.6)	0.748	49 (55.7)	1.2 (0.7–2.0)	0.596	61 (69.3)	0.7 (0.4–1.3)	0.279	46 (52.3)	8.0 (4.1–15.9)	<0.001
Alcohol												
Yes	63 (61.8)	1.1 (0.7–1.7)	0.764	56 (54.9)	1.3 (0.8–2.1)	0.213	70 (68.6)	0.5 (0.3–0.9)	0.017	80 (78.4)	82.1 (38.9–173.2)	<0.001
No	170 (60.1)	1.0		135 (47.7)	1.0		227 (80.2)	1.0		12 (4.2)	1.0	
Duration of disease												
> 5 years	133 (61.0)	1.1 (0.7–1.6)	0.729	108 (81.7)	2.0 (1.2–3.3)	0.004	175 (80.3)	1.4 (0.9–2.3)	0.143	48 (22.0)	0.9 (0.6–1.5)	0.724
≤ 5 years	103 (59.2)	1.0		83 (68.8)	1.0		116 (73.9)	1.0		37 (23.6)	1.0	
Obesity												
Obese, BMI ≥ 30 kg/m^2^	58 (67.4)	1.5 (0.9–2.4)	0.136	46 (53.5)	1.2 (0.8–2.0)	0.414	65 (75.6)	0.9 (0.5–1.6)	0.696	15 (17.4)	0.6 (0.3–1.1)	0.111
Not obese, BMI‹30 kg/ m^2^	175 (58.5)	1.0		145 (48.5)	1.0		232 (77.6)	1.0		77 (25.8)	1.0	
CKD/KDIGO status												
Stages 3,4 and 5	86 (57.3)	0.8 (0.5–1.2)	0.307	91 (60.7)	2.1 (1.4–3.2)	0.001	116 (77.3)	1.0 (0.6–1.7)	0.943	38 (25.3)	1.1 (0.7–1.8)	0.597
Stages 1 and 2	147 (62.6)	1.0		100 (42.6)	1.0		181 (77.0)	1.0		54 (23.0)	1.0	

In this logistic regression, the variables were independently analyzed for the separate cardiovascular risk factors but presented on a single table. The Odds Ratios are unadjusted

There was predominance of female subjects at 65.5%, with mean age 62.1 (12.0) years, younger than the males with 65.7 (11.8) years. The females had a relatively modest formal education, where about 80% of them had either no education or primary level (less or equal to 7 years in school) compared to 54.9% of the males with the same.

Almost half of our study population, 49.6% had hypertension and 69.4% of them were on either Angiotensin Receptor Blocker (ARBs) or Angiotensin Converting Enzyme Inhibitors (ACEis), 63.0% on Calcium channel blockers (CCBs) and 47.8% on a diuretic. Note that there were patients on combinations, CCBs with either ARBs or with ACEis or with diuretics. There were no gender differences.

Regarding treatment of hyperglycaemia, the majority, 68.1% of the study patients were on oral agents only and 12.0% were using combined oral agents with insulin. Just 17.1% were on insulin-only therapy. Glycaemia control was optimal in 39.5%.There were no significant differences in quality of glycaemic control between males and females.

Concerning lipid profile, most patients, 79.2% had normal HDL, 54.5% had high triglycerides and 77.1% had high LDL-cholesterol. Females had higher serum levels of LDL–Cholesterol and total cholesterol than the males. Thirty two (32.7%) percent had albuminuria**.**

Using the body mass index, 40% were overweight (BMI 25.0–29.9 kg/m^2^) while 22.3% were obese (BMI ≥ 30.0.kg/m^2^). Other measures of obesity of waist-hip ratio and waist circumference gave different prevalence of obesity, 92.7% and 58.2% respectively. The body habitus of high waist circumference meant more abdominal emphasis in most of the study subjects. The physical activity of the study subjects was not quantified in this study.

## Discussion

The burden of type 2 diabetes in sub-Saharan Africa is rising and expected to multiply further, [7] consequently their health sector will face an increasing case-loads of cardiovascular morbidities attributable to diabetes in years to come. The objective of this study was to audit the care of patients with type 2 diabetes attending an out-patient clinic in a public hospital.

There were more females, 65.5% in our study, probably reflecting gender-related health-seeking behavior in Kenya because prevalence of type 2 diabetes in Kenya, like the study of Ayah R. and Wanjiru R. et al., in Kibera, Nairobi [12], did not show any gender difference in prevalence of diabetes.

Our patients were mainly from rural dwelling, had low formal education where only 27.8% had attained secondary education and above, in favour of males. The generation of our study population, mean age of 63.3 years, was born in the pre-independent Kenya, when most people did not attend school. Although most of them lacked formal education, they reported self-employment with relative socio-economic stability.

Social determinants of disease in people are important. Socio-economic stability notwithstanding, formal education has a bearing on health literacy. A strong association between low formal education and worse health literacy has been demonstrated in some studies [13, 14], which may translate into poor health status [15]. Socio-economic position influences access to care, healthcare behaviour and processes of care [16]. Over 86% of our patients had visited the clinic 3–4 times in the previous 12 months, meaning they had access to care. It is uncertain how many visits to healthcare provider would be sufficient in a 12-month period in chronic care but the hospital’s capacity to offer quality healthcare also counts. Overall, this study registered low proportions of subjects with desired levels of control of: glycaemia of HbA1c ≤7% at 39.5%, Blood pressure < 140/90 mmHg was 23.4%, and Low density lipoprotein cholesterol (LDL-C), ≤2.0 mmol/L was 22.9%. Access to care is better in the County hospitals but the quality of care still falls below expectations. The SMBG is curtailed by frequent changes in glucometer types and their strips from suppliers [17].

The mean HbA1c was 8.2%, with a predominance of poor glycaemic control. The study patients had had diabetes for more than 5 years (mean duration of diabetes of 9.4 years), but only 29% were on insulin therapy, either in combination or as sole therapy, suggesting that the glucose-lowering treament may not have been intensified. Clinical inertia [18] of care providers and poor adherence to therapies by patients [19, 20] are documented contributors to poor glycaemic control amongst patients with diabetes. We found no predictor of glycaemic control amongst the factors analyzed. In our local context, poor adherence is usually occasioned by circumstances of lack of medications in the public hospital and inability of the patients to afford them elsewhere in private pharmacies [17]. Though we did not assess adherence, it was evident that some of our patients were not taking medications prescribed but not available/dispensed in the hospital pharmacy.

Almost half, 49.6% of the study subjects had hypertension, only 33.4% of the treated patients were on target at the time of evaluation. The subjects with hypertension were older (64 yrs. versus 58 yrs), had diabetes for longer and a significant proportion at higher stages (3, 4, 5) of CKD than those without hypertension. The mean systolic and diastolic blood pressures were 143.6 mmHg and 81.4 mmHg respectively. Over two-thirds, 69.0% were on ACEIs/ARBs, including the subjects that were not on blood pressure targets. It is noteworthy that 66.7% of patients on ACEi/ARBs had no albuminuria but 33.3% on similar treatment had albuminuria, OR (95%CI, 0.7–1.7), *p* = 0.703, suggesting no benefit was conferred in mitigating albuminuria. However, we did not ascertain duration of use, doses of and adherence to ACEi/ARB treatment. This was a point evaluation therefore it requires cautious interpretation. Our patients were also using loop-diuretic (Furosemide), α-methyldopa and hydralazine because they are relatively cheaper than the desired medications. Gill G., et al. [21] reported that such situations also occur when healthcare providers were not aware of the alternative choices or when the hospital system is not stocking what is appropriate. Hypertension drives kidney disease, it markedly increases the odds of having CKD stages 3–5, more so in the older age-group, above 50 years as seen in our patients. That hypertension is associated with declining eGFR has been observed, [22–24], is a causative factor of stroke [25, 26] and acute coronary syndromes in patients with type 2 diabetes. [27, 28] These emphasize the importance of hypertension control in type 2 diabetes. Framingham study demonstrated that an increment of 20 mmHg in systolic or 10 mmHg in diastolic blood pressure doubled the risk of adverse cardiovascular outcomes across the entire range of blood pressure in people between the ages of 40 to 70 years [29]. Most, 76.6%, of our patients had blood pressures above treatment targets putting them at high risk of cardiovascular events.

Out of 77.1% with high LDL-C, eligible for treatment, only 12.5% of our patients were on statin therapy. The females, subjects not on statins and those who were employed had higher serum levels of LDL-cholesterol. The females were also more obese and had higher LDL-C levels than the males in this study. Their mean age of 63 years is post-menopausal, and menopause is associated with increased serum LDL-C [30].

High serum LDL-C levels is often asymptomatic, therefore not surprising that low uptake of statins for primary prevention of cardiovascular events is reported [31, 32]. The reasons advanced to explain low uptake of statins translate to the providers who may not accurately estimate cardiovascular risk, fail to estimate the risk or simply fail to implement guidelines, whose recommendations include periodic assays of serum lipids [32]. We did not determine fidelity to guideline implementation, if there was any in this clinic, but we ascertained that lipid assay was not routine in the hospital, attesting to insufficient laboratory support to clinical care.

At the time of evaluation of the five (5) cardiovascular risk factors, namely, poor glycaemic control, hypertension, high LDL-C, cigarette-smoking and obesity, 43.1% of the subjects had any two (2) risk factors under optimal control. Only 20.3% subjects had the three (3) main risk factors of hyperglycaemia, hypertension and high LDL-C simultaneously in optimal control at the time of this study, which was rather dismal. The care context observed during the study was simplistic in organization, insufficient in staff empowerment, had sub-optimal quality of diabetes self-management education and inadequate laboratory support to care therefore not supportive of multiple risk factor reduction. Beran D., et al. [33] and Whiting D., et al. [34] in studies also in sub-Saharan Africa, made similar observations.

Evidence has shown that intensified cardiovascular risk factor control in patients with type 2 diabetes, either as single or double risk factor reduction, significantly mitigates morbidity and mortality [35–40]. However, Drake et al. [41], re-analyzing the ACCORD study, demonstrated that over 30% of the study subjects who had not achieved treatment targets were due to clinical and demographic reasons that highlighted the inherent challenges underlying the care of diabetes. Regarding our patients, it is also probable, amongst other reasons that we did not study, that treatment intensification and risk stratification of patients were not done.

Reducing multiple cardiovascular risk factors simultaneously in subjects with type 2 diabetes has already been demonstrated as a feasible strategy [42–45], but with overwhelming support to systems of care during the clinical trials. Those levels of gain have not been replicated in routine care. For instance, lowering SBP by 4 mmHg, LDL-C by 1 mmol/L and HbA1c by 0.9% reduced cardiovascular events by 12.5%, 8.2% and 2.9% respectively [46–48], additional to other good lifestyle practices over time. Wong N, et al., further showed that reducing these risk factors simultaneously in the patients with type 2 diabetes incrementally lowered cardiovascular event rates [49]. Those three main risk factors drive cardiovascular events and their control confers relative cost-benefits on diabetes-associated outcomes. Self-care in patients with diabetes should be sufficient to address the multiple risk factors that one may have. However, this is often a pipedream. Studies in sub-Saharan Africa, like Ethiopia [50, 51] and Nigeria [52] reported poor self-care behaviors, including poor adherence to therapies. One quarter of our patients were cigarette-smokers, mainly men who under-reported the habit. Cigarette-smoking is an important cardiovascular risk whose remedy is reported to be challenging in Africa [53, 54]. Sub-Saharan Africa is not achieving goals of control of non-communicable diseases [55]. From this study, it is probable that social determinants may be playing a big role, especially the low formal education, high unemployment and consequent low economic capacity.

In the sub-Saharan Africa, the challenges of diabetes care occur at both individual level and healthcare systems that favor acute care over chronic care. The national policies are skewed and capacities in disposable resources attenuated. These factors conspire to deter achievement of optimal control of the different cardiovascular risk factors simultaneously [56]. It is imperative that care strategies that achieve simultaneous control of cardiovascular risk factors to targets may be the main challenge or probably the missing link between the clinical care we offer and desired gains we should receive. Compared to instruments of care and new agents, delivery of care may be the bigger problem in sub-Saharan Africa. We recommend innovative designs of health systems and care delivery which would comprise reliable financing, functional internal and external system linkages, efficient teams with appropriate care attitudes and stewardship. These should form the next frontiers of operations research to inform clinical management of patients with type 2 diabetes who access care.

## Conclusion

We found high prevalence of cardiovascular risk factors in our patients who attended the public hospital clinic and they were inadequately controlled. The reasons settle down to sub-optimal quality of care because of modest socio-economic status of most patients, insufficient resources to support clinical staff and laboratory to meet care requirements. This put a large proportion of the patients at risk of cardiovascular events with potentially obvious challenges in their care. This is a common picture in many public health facilities that the majority of our patients visit for both acute and chronic care.

## Abbreviations

CKD/KDIGO: Chronic Kidney disease/Kidney Disease Improving Global Outcomes
IQR: Interquartile range
